# A novel germline mutation of *TP53* with breast cancer diagnosed as Li–Fraumeni syndrome

**DOI:** 10.1186/s40792-022-01546-y

**Published:** 2022-10-11

**Authors:** Masaya Kai, Makoto Kubo, Sawako Shikada, Saori Hayashi, Takafumi Morisaki, Mai Yamada, Yuka Takao, Akiko Shimazaki, Yurina Harada, Kazuhisa Kaneshiro, Yusuke Mizuuchi, Koji Shindo, Masafumi Nakamura

**Affiliations:** 1 Department of Surgery and Oncology, Graduate of School of Medical Sciences, Kyushu University, 3-1-1 Maidashi, Higashi-ku, Fukuoka, 812-8582 Japan; 2 Department of Clinical Genetics and Medicine, Kyushu University Hospital, Fukuoka, Japan

**Keywords:** Li–Fraumeni syndrome, *TP53* pathogenic variant, Breast cancer, Hereditary cancer, Genetic medicine, Genetic testing, Multi-gene panel assay

## Abstract

*TP53* is a tumor suppressor gene and, when dysfunctional, it is known to be involved in the development of cancers. Li–Fraumeni syndrome (LFS) is a hereditary tumor with autosomal dominant inheritance that develops in people with germline pathogenic variants of *TP53*. LFS frequently develops in parallel to tumors, including breast cancer. We describe a novel germline mutation in *TP53* identified by performing a multi-gene panel assay in a breast cancer patient with bilateral breast cancer.

## Background

*TP53* has been known as a tumor suppressor gene for three decades and plays a role in cell cycle regulation, including DNA repair, cell growth arrest, and induction of apoptosis [1]. The *TP53* gene consists of 393 amino acids [2], is located on the short arm of chromosome 17 (17p13.1), and is evolutionally preserved. Mutations in *TP53* are most frequently detected in malignant tumors [3].

Li–Fraumeni syndrome (LFS) is an autosomal dominant hereditary disorder that occurs in patients with germline mutations in *TP53* gene [4]. The diagnosis of LFS can be established if the patient conforms to the classical diagnostic criteria and is family based on the germline variant of *TP53*. When LFS is clinically suspected, it is recommended to go for genetic testing of *TP53*. LFS is considered a syndrome of high penetrance, with reports of a lifetime cancer risk of more than 70% in men and 90% in women [5, 6] and an 80% risk of cancer by age 70 [7]. Recently, germline pathogenic variants of *TP53* have been identified, and diagnosed LFS may not meet the classical LFS or Chompret criteria, suggesting that LFS penetrance may be overestimated [8]. Patients frequently develop LFS-related tumors, such as breast cancer, osteosarcoma, soft tissue sarcoma, cerebral tumor, and adrenal cortical carcinoma, or sometimes have many types of malignancies simultaneously, including hematological and pediatric cancers [9]. It has been suggested that variant types of *TP53* may influence clinical features; however, to date, this has not been fully elucidated. In this study, we report a novel *TP53* germline pathogenic variant identified using a multi-gene panel assay in a patient with metachronous bilateral breast cancer who had a family history of LFS-related tumors.

## Case presentation

**Fig. 1:**
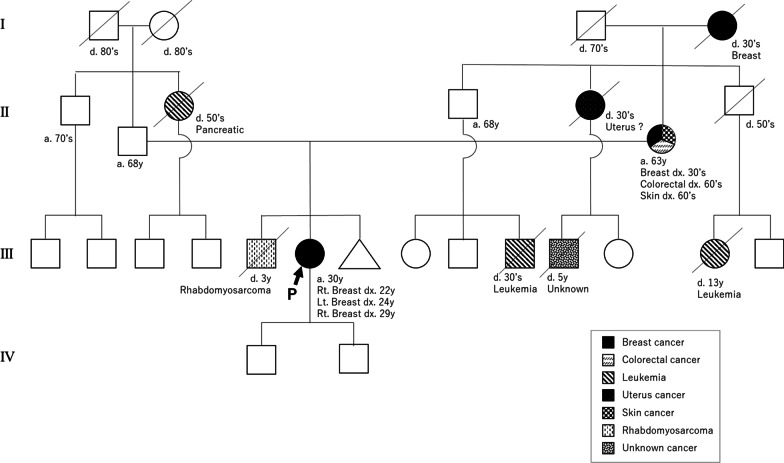
Pedigree of cancer history. The patient is presented as the first proband (P) in the third generation. The patient’s mother was affected by breast, colorectal, and skin cancer, and was positive for the germline *TP53* mutation. The patient’s brother died of rhabdomyosarcoma at the age of 3

The patient was a woman who was first diagnosed with cancer in her right breast in her 20s. She underwent right breast-conserving surgery and a sentinel lymph node biopsy was assessed. The pathological diagnosis was ductal carcinoma in situ, pTisN0M0 stage 0, with radiation therapy of 50 Gy in 25 fractions of the right breast but without any adjuvant systemic therapies. Two years after her first diagnosis, she was diagnosed with cancer in her left breast and underwent nipple-sparing mastectomy with sentinel lymph node biopsy and tissue expander insertion in her left breast. The pathological diagnosis was ductal carcinoma in situ and pTisN0M0 stage 0, and the patient did not undergo radiation or adjuvant systemic therapies. Seven years after her initial diagnosis, a recurrent tumor was found in the right breast. Core needle biopsy showed invasive ductal carcinoma, which was ER 3%, PgR 0%, and HER2 negative, and the subtype was almost triple-negative breast cancer. She received preoperative systemic therapy with dose-dense AC (doxorubicin and cyclophosphamide) followed by dose-dense paclitaxel. She underwent nipple-sparing mastectomy with sentinel lymph node biopsy and tissue expander insertion in the right breast. The final pathological diagnosis was microinvasive carcinoma (< 1 mm) and the therapeutic effect was grade 2b. After surgery, she received capecitabine for 6 months as adjuvant systemic therapy. The patient had no remarkable medical history including neoplasms. Her family history was as follows: her older brother had rhabdomyosarcoma when he was 3 years old, and her mother had breast cancer in her 30s, colon cancer in her 60s, and skin cancer at 63 years of age. The details of other family trees are shown in Fig. 1.

**Fig. 2:**
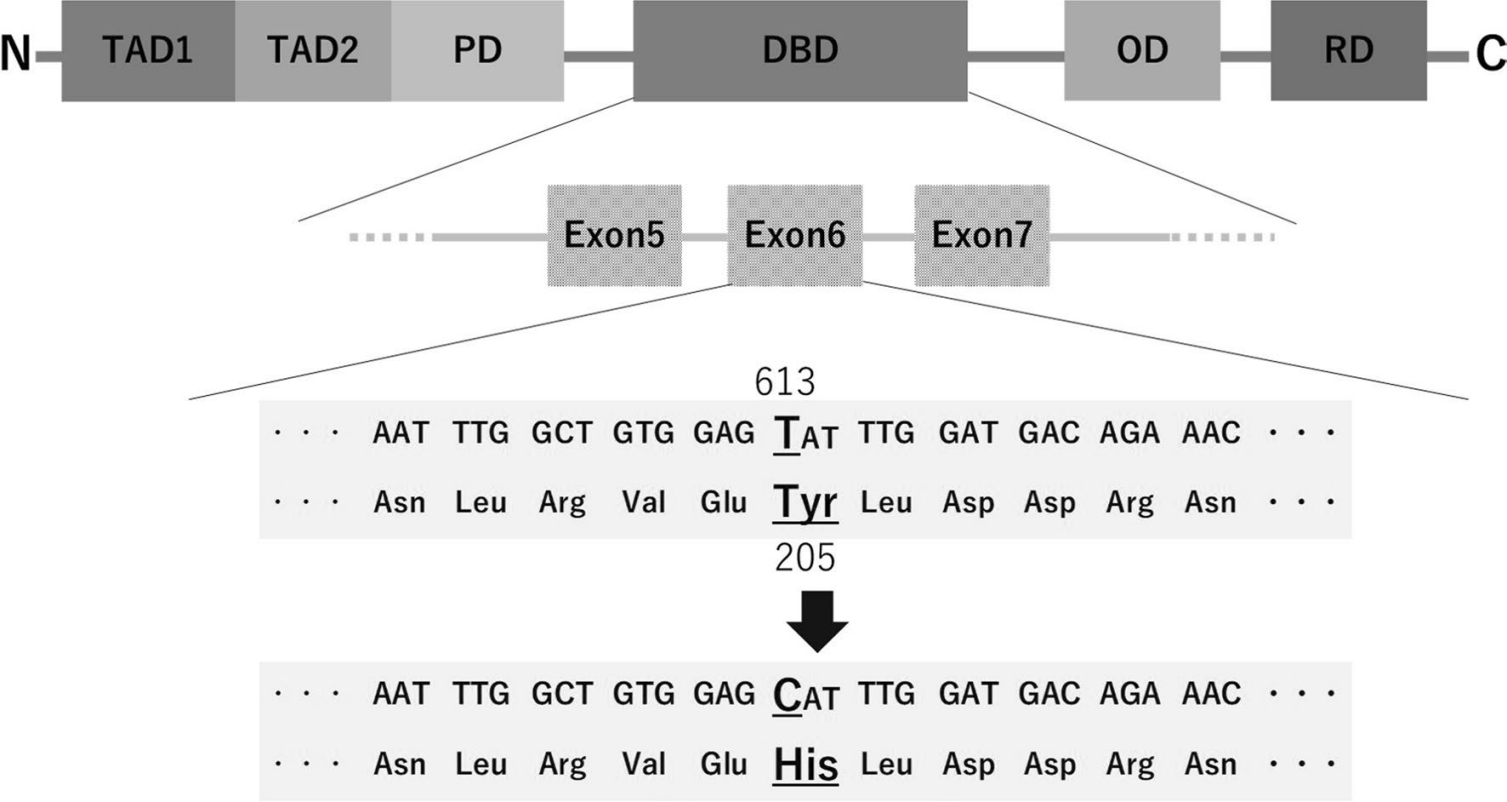
Schematic diagram of the *TP53* gene. 613T>C (p.Tyr205His) is highlighted by underbar. *TAD* transactivation domain, *PD* proline-rich domain, *DBD* DNA binding domain, *OD* oligomerization domain, *RD* lysine-rich regulatory domain

Based on her past breast cancer and family history, she was initially suspected of having LFS because the patient met the classical diagnostic criteria for LFS since her older brother was defined as the proband. This case also met the Chompret criteria [10]; that is, the patient had breast cancer before the age of 46 years and had a second-degree relative with an LFS tumor younger than 56 years of age. Based on these findings, this case was strongly suspected to be LFS and genetically tested for *TP53*. However, the hereditary breast and ovarian cancer (HBOC) syndrome has not been ruled out. Therefore, the comprehensive analysis of hereditary tumor-related genes is thought to be useful. She underwent a multi-gene panel assay with Myriad myRisk^®^ hereditary cancer genetic testing (Myriad Genetics, Inc., Salt Lake City, UT, USA). A genetic variant was detected, *TP53* c.613T>C (p.Tyr205His) (Fig. 2). However, this variant has not been reported as pathogenic in germline but somatic mutation, and the evaluation of multi-gene assay was “special interpretation”. The suspected deleterious variant was consistent with a heterozygous germline mutation, which would cause the inherited cancer condition LFS; however, some studies have shown that there are cases where *TP53* mutations detected through genetic testing are not present in the germline but rather arise as somatic mutations that may not be present in all tissues.

Therefore, at that time, the patient was not diagnosed with LFS. Next, we conducted further genetic analysis of her parents, single-site gene analysis with Myriad myRisk^®^ hereditary cancer genetic testing. Although it was very hard to access her parents because they lived far away and separately, they finally received the genetic test with informed consent. Her mother had the same variant of *TP53* c.613T>C (p.Tyr205His), but her father had no clinically significant mutations. According to the ACMG_AMP guideline [11], the patient was evaluated as “likely pathogenic”. Ten years after her first diagnosis of breast cancer, she underwent gastroscopy for gastric discomfort and was diagnosed with gastric cancer. In this case, the diagnosis of LFS was confirmed based on the results of genetic testing of the patient with heterogeneous bilateral breast cancer and her mother. At our hospital, the Department of Clinical Genetic Medicine, in collaboration with the Department of Breast Surgery, Gynecology, Plastic Surgery, and Radiology, has discussed and established a system to avoid radiation exposure, and establish surveillance and psychological intervention for the patient and her family. Surveillance is performed mainly by whole-body MRI and abdominal ultrasonography, and comprehensive support is provided.

## Discussion

LFS occurs in patients with *TP53* germline pathogenic variants [4]. The frequency of pathogenic variants in the general population is reported to be one 5000–20,000th [12, 13]. LFS-related tumors, such as breast cancer, osteosarcoma, soft tissue sarcoma, brain cancer, and adrenal cancer, occur at a high rate from a young age, and ones such as hematologic malignancy, epithelial cancer, and pediatric cancer can also occur. Approximately half of these cases occur simultaneously or asynchronously in multiple malignancies. Although the IBTR ipsilateral breast tumor recurrence rate is 3.9% at 10 years after whole-breast irradiation [14], radiation therapy in LFS may increase the risk of secondary malignancies [15]. Similarly, it has been suggested that chemotherapeutic agents, especially alkylating agents, may influence the development of hematologic malignancies [16]. Therefore, it is recommended that such treatment and examination should be avoided whenever possible.

It has been suggested that the types of *TP53* pathogenic variants and factors that modify the function of *TP53* affect its clinical features, but this is not certain at this time. LFS can be diagnosed by the detection of a pathogenic variant of *TP53* [4]. Some individuals who do not have a *TP53* pathogenic variant may meet the classical LFS diagnostic criteria and receive that diagnosis. In addition, the Chompret criteria [10] are widely used as the basis for possible LFS and genetic testing for *TP53*. Mutations in *TP53* c.613T>C (p.Tyr205His) were also detected. Notably, this gene mutation has been reported as a somatic mutation, but has never been reported as a germline mutation, which is annotated as “special interpretation” at that time. This mutation does not indicate a definitive diagnosis of LFS because there is a possibility of contamination, such as detection according to the treatment history of chemotherapy or the presence of tumor cells in the blood. An experimental study in yeast showed that this variant impairs the transcriptional transactivation activity of the TP53 protein [17] and disrupts its p.Tyr205 amino acid residue. Other variants that disrupt this residue have been observed in individuals with *TP53*-related conditions [18–20], suggesting that it is a clinically significant residue. As a result, variants that disrupt this residue are likely to cause disease. In summary, the available evidence is insufficient to determine the role of this variant in various diseases. Therefore, it has been classified as a variant of uncertain significance. Studies conducted in human cell lines indicated that this alteration is deficient in growth suppression [21, 22]. This amino acid position is highly conserved among vertebrates. Additionally, according to in silico analysis, this variant was predicted to be deleterious. Based on the majority of evidence available to date, this variant is likely to be pathogenic. To assess this, testing of close relatives has been suggested to be useful, and genetic testing of the parents has been performed. Because the mother had the same *TP53* germline mutation, the patient was applied to PM2, PM5, PP1, PP2, PP3, PP4, and evaluated as “likely pathogenic”, according to the ACMG_AMP guidelines [11]. Segregation studies are necessary to provide stronger evidence to support the pathogenicity of this variant. However, a segregation study for positive results was not possible in this family because all affected individuals, except the tested person and her own mother, have already died.

## Conclusion

We reported a *TP53* germline mutation*,* c.613T>C (p.Tyr205His) as pathogenic in the patient with metachronous bilateral breast cancer using a multi-gene panel assay myRisk^®^ for hereditary cancer. Genetic testing of relatives was essential for diagnosing pathogenic mutations precisely as the myRisk^®^ report suggested at that time.

## Data Availability

Please contact the corresponding author for data requests.

## Abbreviations

LFS: Li–Fraumeni syndrome
ER: Estrogen receptor
PgR: Progesterone receptor
HER2: Human epidermal growth factor receptor 2
VUS: Variant of uncertain significance
MRI: Magnetic resonance imaging
